# The Role of the Environment and Exposome in Atopic Dermatitis

**DOI:** 10.1007/s40521-021-00289-9

**Published:** 2021-05-21

**Authors:** Nicholas Stefanovic, Alan D. Irvine, Carsten Flohr

**Affiliations:** 1 St James’ Hospital, Dublin, Ireland; 2 Department of Paediatric Dermatology, Children’s Health Ireland at Crumlin, Dublin, Ireland; 3 National Children’s Research Centre, Crumlin and Clinical Medicine, Trinity College Dublin, Dublin, Ireland; 4 Unit for Population-Based Dermatology Research, St John’s Institute of Dermatology, Guy’s & St Thomas’ NHS Foundation Trust and King’s College London, London, UK

**Keywords:** Atopic dermatitis, Atopic eczema, Eczema, Exposome, Environment, Microbiome

## Abstract

**Purpose of review:**

Atopic dermatitis (AD) is a chronic inflammatory skin disorder affecting up to 20% of children and up to 5% of adults worldwide, contributing to significant disease-related morbidity in this patient cohort. Its aetiopathogenesis is underpinned by multiple factors, including genetic susceptibility, skin barrier defects, a skewed cutaneous immune response and microbiome perturbation in both the skin and the gut. In this review, we aim to examine the biological effects of key environmental exposures (the sum of which is termed the “exposome”) at the population, community and individual levels in order to describe their effect on AD pathogenesis.

**Recent findings:**

It is now understood that as well as considering the type of environmental exposure with regard to its effect on AD pathogenesis, the dosage and timing of the exposure are both critical domains that may lead to either exacerbation or amelioration of disease. In this review, we consider the effects of population-wide exposures such as climate change, migration and urbanization; community-specific exposures such as air pollution, water hardness and allergic sensitisation; and individual factors such as diet, microbiome alteration, psychosocial stress and the impact of topical and systemic therapy.

**Summary:**

This review summarises the interaction of the above environmental factors with the other domains of AD pathogenesis, namely, the inherent genetic defects, the skin barrier, the immune system and the cutaneous and gut microbiota. We specifically emphasise the timing and dosage of exposures and its effect on the cellular and molecular pathways implicated in AD.

## Introduction

Atopic dermatitis (syn. atopic eczema) (AD) is a chronic inflammatory skin disorder affecting up to 20% of children and 10% of adults worldwide, with inter-regional variability both between and within countries [1–3]. It is associated with a significant symptom burden, including pruritus, pain and sleep disturbance. A negative impact on self-esteem and educational/work performance are features associated with an adverse impact on an individual’s quality of life [4]. Furthering our knowledge of the mechanisms underpinning the aetiopathogenesis of AD is imperative for amelioration of symptoms and the institution of targeted prevention and treatment approaches. Current understanding of AD pathogenesis points towards a sophisticated interplay between a genetically determined skin barrier defect, innate and adaptive immune dysregulation with a T-helper 2 cell (T_H_2)–dominant phenotype, dysbiosis of cutaneous and gut microbiota, as well as environmental risk factors [5, 6].

**Fig. 1:**
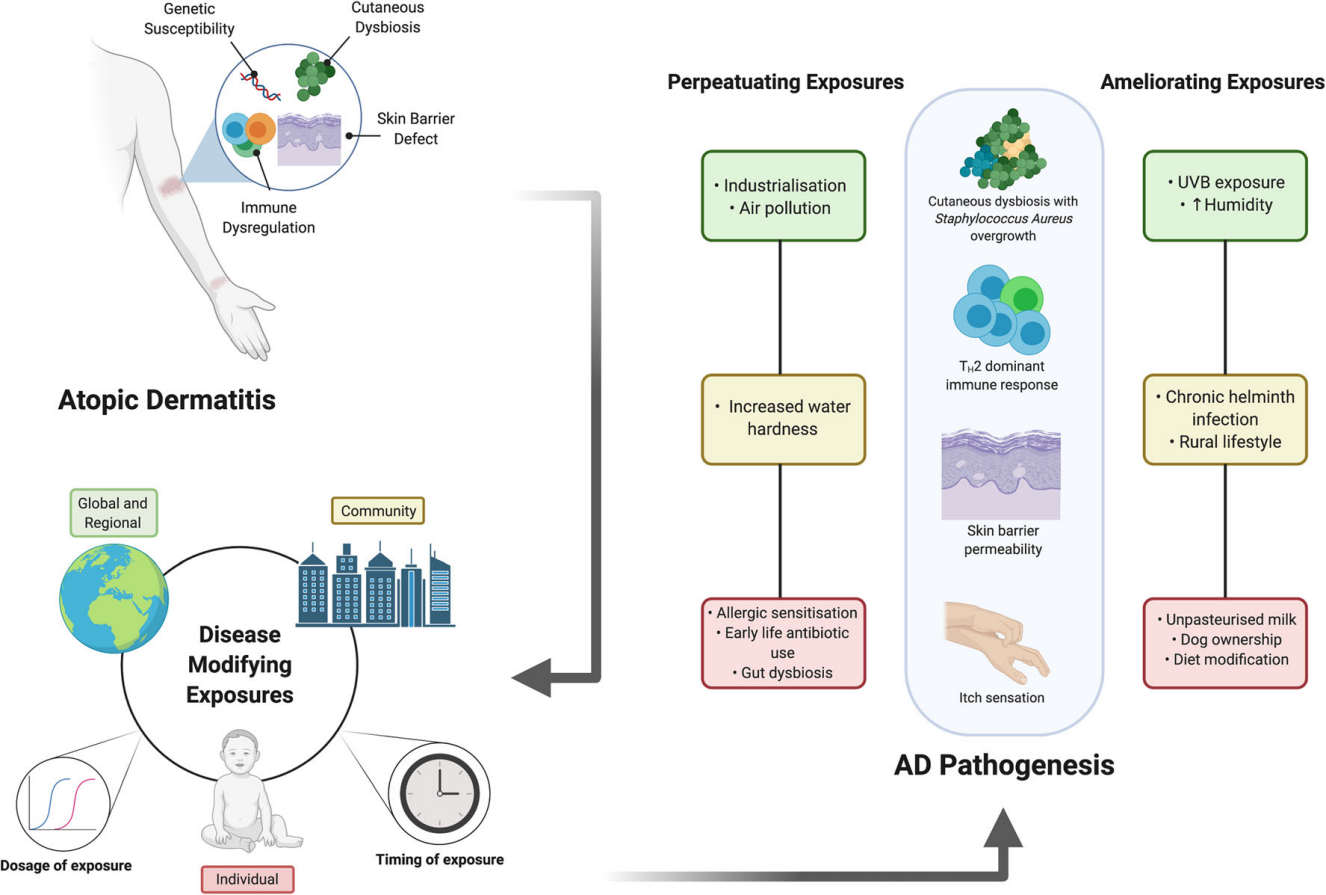
**Graphical abstract**. Atopic dermatitis (AD) is a chronic inflammatory condition, affecting up to 20% of children and up to 5% of adults worldwide. The pathogenesis of AD is multifactorial, involving genetic susceptibility, skin barrier dysfunction and inflammation as well as microbial dysbiosis. The course of the disease can be modified by external exposures that interact with the above pathogenic pathways and the biological response to such exposures is termed an individual’s “exposome”. Disease-modifying exposures are common to populations, communities and individuals. An exposomal approach to scientific enquiry enables us to identify the timing and dosage of such exposures in an attempt to either ameliorate disease or its exacerbation, merging the domains of epidemiology with cellular and molecular biology. In this narrative review, we discuss the key external exposures pertinent to AD pathogenesis at the population, community and individual levels and examine their effect on the known perturbations in biologic pathways pertinent to AD

The biological response to the sum of environmental factors an individual is exposed to from conception to death is termed the exposome [7]. It merges the domains of epidemiology, molecular and cellular biology in order to highlight the links between genetics, immunology, microbiology and the environment as it relates to a particular disease. Identifying the pertinent exposome highlights the pathways via which the human and natural environment contribute to disease pathophysiology [8•]. Exposomal enquiry enables modification of pathogenic pathways by altering degrees and timing of exposure to key environmental factors thereby influencing the disease course. Recent advances in molecular biology techniques and a shift in conceptualization has enabled us to broaden our understanding of the biomarkers of disease and the role of distinct environmental factors in pathophysiology [9]. As a consequence, we can measure and analyse the impact of environmental factors common to wider populations (e.g. climate change, migration, urbanization), local communities (e.g. air pollution, water hardness, allergen sensitisation) and individuals (e.g. diet, individual level microbiome alteration, impact of topical and systemic therapy) as they pertain to the aetiopathogenesis of AD. Given the high population prevalence, significant symptom burden, accessibility of the skin as an organ and the resulting exposure to the environment, AD is uniquely poised for exposomal intervention [10] (Fig. 1—Graphical abstract)**.**

There is a well-established association between AD and atopic comorbidities, sometimes called the “atopic march”, though the relationships between and timing of onset of atopic comorbidities are more complex and many different patterns exist [11••, 12]. Nonetheless, an enhanced understanding of environmental factors contributing to the pathogenesis of AD in early life would potentially enable early regulation of the systemic immune response, allergic sensitisation and microbiome dysregulation in an effort to curb development of further atopic comorbidities [13]. In this review, we consider the effects of environmental factors on the skin barrier, immune system and the microbiome in the quest to identify prevention and treatment pathways for potential intervention.

## Global population factors

The exposome has been conceptualized as the sum of external factors (both specific and non-specific) and internal factors (individual level variables such as diet, and by-products of metabolism acting on an individual’s internal cellular milieu) that affect an individual from conception to death [7]. We propose an alternative model for identifying and modifying environmental exposures based on interventions focused on national and international populations, smaller communities within specific regions and, indeed, individuals themselves.

Differences in lifestyle and population factors associated with industrialization have been linked with a higher prevalence of AD symptoms in the International Study of Asthma and Allergies in Childhood (ISAAC) Phase 1 [14]. Analysis of longitudinal data from ISAAC Phase 3 demonstrated no increase in prevalence in countries that are typically classed as high prevalence, whilst a rise in disease prevalence was demonstrated in low-income settings such as Asia and Latin America, coinciding with changes in lifestyle linked to an increase in industrialization [2].

### The role of UV exposure

One of the mechanisms potentially responsible for increased prevalence of AD in regions that undergo industrialization may pertain to environmental factors related to global climate change, as increased greenhouse gas emission and rising global temperatures lead to perturbations in humidity and atmospheric UV radiation levels [15]. AD is commoner in areas of low relative humidity, low outdoor temperatures, low levels of ultraviolet (UV) light exposure and increased use of central heating [16••, 17, 18]. Low-dose UVB exposure has been demonstrated to accelerate epidermal skin barrier recovery, synthesize antimicrobial peptides and lipids as well as increase expression of 1-alpha hydroxylase in mouse models [19]. In the presence of UV light, the filaggrin breakdown product (FBP) trans-urocanic acid is converted to the immunosuppressive cis-urocanic acid, which in mouse models has been demonstrated to reduce epidermal inflammation with a lower mast cell infiltrate and reduced serum IgE concentration [20]. The cutaneous microbiome is altered by UV radiation in a dose-dependent manner and may contribute to reduced dysbiosis and colonization by superantigen-producing species, such as *Staphylococcus aureus,* which consequently leads to an influx of T_H_2 cells, IL-4 and IL-13 production and further recruitment of histamine-producing mast cells and eosinophils [21, 22•]. Recent evidence also points towards a role of cutaneous UVB exposure in increasing gut microbiome diversity via increased hydroxylation of vitamin D in the skin-gut microbiota axis [23]. Of particular significance, bacteria from families *Lachnospiraceae* and *Ruminococcus* were more abundant in faecal samples of study subjects following low-dose cutaneous UVB exposure. The relative deficiency of the above bacterial families in faecal samples from infants with AD has been demonstrated to be associated with reduced expression of host immunity-regulating genes [24]. Low relative humidity in regions of high AD prevalence likely exacerbates the above pathways via reduction of filaggrin expression and enhanced deimination of filaggrin to natural moisturizing factor (NMF), further perturbing the skin barrier [25, 26]. The above findings may at least partially explain the increased susceptibility to AD flares during cold and dry periods of the year [18].

### The role of air pollution

Alongside climate change, increased levels of air pollution have been demonstrated to correlate positively with AD symptoms [27]. Air pollutants comprising volatile organic compounds (VOC), particulate matter (PM), traffic related air pollution (TRAP) and tobacco smoke have been demonstrated to have a detrimental effect on skin barrier integrity via generation of reactive oxygen species (ROS), as well as epigenetic modification of the immune system in utero, polarizing the adaptive immunity towards a T_H_2 phenotype. This subsequently predisposes infants towards a proinflammatory cutaneous immune profile in the early postnatal period [28, 29]. Skin barrier disruption is likely facilitated via reduced expression of epidermal structural proteins (filaggrin, cytokeratin, E-cadherin) in response to PM application, whilst exposure to VOC has been demonstrated to increase transepidermal water loss (TEWL) via a yet unidentified mechanism [30, 31]. The immune system is likely primed via activation of aryl hydrocarbon receptor (AhR) and nuclear factor kappa B (NFkB) signalling, leading to granulocyte infiltration, perpetuating inflammation [27]. AhR signalling has also been demonstrated to induce hypersensitivity to pruritus via enhanced production of the neurotrophic factor artemin in a mouse model [32]. Exposure to PM may further exacerbate AD by leading to cutaneous dysbiosis, as exposure has been demonstrated to enhance cutaneous *S. Aureus* colonization in animal models [33]. The evidence for tobacco smoke pinpoints to an interplay between epigenetic mechanisms occurring in utero, whereby exposure in the third trimester of pregnancy is correlated with an AD phenotype in early infancy, likely through epigenetic priming of the immune phenotype towards a T_H_2-dominated skew, as well as further direct damage to the epidermal proteins in the early postnatal period [34, 35]. The role of air pollution in AD flares has been supported by several epidemiological studies. A large South Korean cohort study demonstrated a positive correlation between exposure to higher annual mean levels of fine PM and NO_2_ and prevalence of AD symptoms [36]. Short-term elevations in fine and coarse PM were demonstrated to induce AD flares. Bakeout of VOCs has been separately shown to be an effective technique in reducing AD symptom burden by minimizing indoor air pollution [37, 38]. Given the links between increasing airborne pollution and AD prevalence, it is pertinent that population-, community- and individual-level interventions are implemented in an effort to prevent the development of AD and ameliorate symptoms. Changes in traffic demand during the COVID-19 pandemic have decreased environmental NO_2_ levels by up to 30% [39]. The effect of this unprecedented environmental change on AD prevalence remains to be studied but may provide further insight on the impact of airborne pollution, particularly TRAP in AD. Given the challenges facing population-level interventions to prevent climate change and worsening global pollution, it is imperative that a multifaceted exposomal approach is taken. Individual-level approaches such as barrier restoration with emollients and antioxidants, as well as personal avoidance of exposure to high levels of pollution (where possible) may be adopted as a complementary strategy [40].

## Community and regional environmental factors

Whilst factors such as urbanisation, pollution and climate change affect populations globally, certain disease-modifying environmental exposures affect specific regions within individual countries and their resident communities. Cross-sectional and cohort studies have demonstrated an increased prevalence of AD symptoms in areas with increased levels of water hardness, although no definitive relationship was reported for chlorine [41, 42]. Data from the Enquiring About Tolerance (EAT) study demonstrated a three-fold increased risk of AD development in infants with *FLG* mutations, as well as a 23.2% increase in TEWL in infants with *FLG* mutations exposed to hard water, even without AD [43•]. Current understanding of the mechanisms underpinning this point towards barrier disruption. Hard water increases epidermal deposition of sodium lauryl sulphate (SLS); a detergent commonly present in commercially available washing products [44]. It is thought that SLS deposition decreases profilaggrin expression, with subsequent reduction in NMF and up-regulation of protease activity [45, 46]. An interventional study is ongoing in the UK, aimed at examining whether installation of a water softener before birth is able to prevent skin barrier breakdown and AD development [47].

Helminth infections are endemic to regions of South America, Africa and Asia. It is postulated that chronic helminthic infection modulates immunity by inducing T_reg_ cells and IL-10 production, thereby dampening the T_H_2-skewed immune response and conferring a degree of protection against atopic disorders [48, 49]. Consequently, upon treatment of the helminthic infection, an atopic phenotype is manifested by the T_H_2-primed immune system. Crucially, the timing of infection and its eradication is important. Evidence from a placebo-controlled trial in Uganda demonstrated increased incidence of AD in offspring of women who received treatment with albendazole in the third trimester of pregnancy, whilst a separate study from Vietnam found no increased prevalence of AD when antihelminthic treatment was instituted in early childhood [50, 51].

## Individual risk factors

Recent data points towards intrinsic inter-individual heterogeneity in AD severity, onset, response to therapy and the implicated molecular mechanisms also known as endotypes [52•]. T_H_2 immune predominance is seen across all endotypes; however, the relative preservation of barrier function and the modulation of additional adaptive immune pathways such as T_H_1, T_H_17 and T_H_22, as well as serum IgE levels, appear to be influenced by age and ethnicity [52•, 53]. Disease progression and the development of atopic multimorbidity are associated with younger age of AD onset, parental history of atopy, filaggrin mutations and allergic polysensitisation, as well as living in an urban environment [12]. An exposomal approach would examine an individual’s biological and chemical exposures at distinct timepoints throughout their life and their concerted effect on immunity, barrier function and the microbiome. Such an approach (although not currently widely employed due to technical limitations) complements current knowledge of AD endotypes, enabling specific intervention as part of a personalised therapeutic approach.

### The role of allergens

A defective skin barrier is thought to be an early initiating factor in the progression to atopic multimorbidity, with earlier age at initial AD diagnosis correlating strongly to food allergen sensitisation [54, 55•]. Inheritance of *FLG* loss-of-function mutations in AD is associated with an earlier age of onset, suggesting a link for gene-environment interaction in allergic sensitisation [56]. Once sensitised, further transepidermal exposure to food and aeroallergens allergens perpetuates inflammation, contributing to chronicity of inflammation. Evidence in support of this theory comes from a study of topical exposure of sensitised individuals to grass pollen, which demonstrated worsening AD symptoms and elevated serum levels of key T_H_2 cytokines following exposure [57]. A recent study by Leonard et al. highlighted four distinct allergen endotypes (food, seasonal, perennial, mixed) in individuals with AD and demonstrated a specific inflammatory signature upregulated in each type [58]. Furthermore, they highlighted an upregulated IgE response to *S. aureus* toxic shock syndrome toxin-1 in subjects displaying perennial and seasonal endotypes, suggesting a link between persistent topical allergen exposure and cutaneous dysbiosis. A systematic review by Tsakok and colleagues linked increasing AD chronicity and severity in infants with food sensitisation and allergy, with AD symptoms commonly preceding those of food allergy, indicating a causal relationship [59]. Epidemiological findings from the Canadian Longitudinal Healthy Infant Study (CHILD) highlighted that infants with AD who were poly-sensitised to multiple airborne and food allergens by 3 years of age were at higher risk of developing allergic comorbidities, compared with infants with AD who were mono-sensitised or non-sensitized [60]. Taken together, the above findings suggest that sensitisation to perennial and seasonal allergens occurs across the skin barrier, can perpetuate AD chronicity and barrier impairment, consequently leading to polysensitisation and the development of allergic multimorbidity.

### Epidermal barrier restoration

Given the ubiquitous prevalence of topical aeroallergens such as grass pollen and house dust mites in the environment, allergen avoidance may not be a feasible strategy for individual-level intervention. An alternative approach aimed at reducing dysbiosis and restoring the epidermal barrier must therefore be adopted. Recent data from the Barrier Enhancement for Eczema Prevention (BEEP) trial of 1394 infants has shown that daily emollient use from birth in the first year of life has not been effective in preventing the development of AD and may indeed confer an increased risk of skin infection, despite being a simple and cost-effective intervention [61, 62••]. The mechanism underpinning this may be attributed to the theory of defective extracellular lipid synthesis and delivery due to an underlying genetic predisposition, leading to increased TEWL and structural perturbation of the “bricks and mortar” of the stratum corneum [63]. As a result, the defective structure enables enhanced permeability to allergens and dysbiotic bacteria, triggering the T_H_2-polarised immune response. Topical emollients form an occlusive barrier on the skin surface and provide symptomatic relief but do not correct the underlying molecular defect. Further investigation into physiologic lipid-based barrier restoration therapy in AD is therefore warranted despite promising early trials [64]. New data from the EAT study suggests that there is a dose-response relationship between emollient application frequency and subsequent development of food allergy (Reference in press, JACI 2020). The mechanism behind this most likely involves transcutaneous sensitisation through regular contact of parents’ hands with their child’s skin, as they apply the emollient. Nevertheless, a positive role of emollients and their therapeutic utility may lie in the regulation of the cutaneous microbiome. The defective skin barrier contributes to an increased stratum corneum pH, which subsequently promotes growth of *S. aureus* and *Streptococcus pyogenes* species, with diminished *Corynebacterium* and *Staphylococcus epidermidis* populations [65, 66]. Emollient therapy has been demonstrated to lower skin pH and restore microbial diversity, particularly with *Streptococcus salivarius* populations [67]. Increased skin pH further impairs the cutaneous barrier in a kallikrein-5-dependent manner, via thymic stromal lymphopoietin 2 secretion and T_H_2 cell chemotaxis, making acid-base balance restoration a lucrative target for therapy [68].

### Modulation of skin and gut microbiota

Skin and gut microbiota are particularly susceptible to external influences and modification at the individual level, given their interface between an individual’s internal milieu and the external environment [69••]. The healthy skin microbiome is topographically diverse and dominated by four main phyla—*Bacteroidetes*, *Firmicutes*, *Actinobacteria* and *Proteobacteria* [6]. The neonatal cutaneous and gut microbiome is influenced by the mode of delivery in the first instance, with caesarean section delivery conferring a microbiome enriched in *Staphylococcus spp.*, whilst infants delivered vaginally have a cutaneous and meconium microbiome colonised by *Lactobacilli* and *Prevotella* species [70]. Following delivery, the skin microbiome is rapidly colonised by commensals, tolerance to which is induced via an influx of T_reg_ cells into the epidermal comparment [71]. In AD, both lesional and non-lesional skin shows reduced commensal diversity and increased abundance of *Staphylococcus* species, in particular *S. aureus* [72]*.* The mechanisms contributing to dysbiosis are multifactorial and include disturbance in skin pH, environmental humidity and temperature changes, as well as other external factors such as frequent antibiotic use alter the composition of skin commensal populations [73]. Consequently, production of antimicrobial peptides (AMP) by commensals such as *S. epidermidis* is reduced, leading to increased colonisation by *S. aureus* [74]*. S. aureus* is a potent inducer of T_H_2 lymphocyte and mast cell chemotaxis, as well as a producer of several toxins and superantigens that have a directly damaging effect on keratinocytes and the epidermal barrier [75•, 76•]. The consequent inflammation perturbs the skin barrier and inhibits the production of AMPs, facilitating an enhanced cycle of dysbiosis [75•, 77]. Perturbation of the skin barrier enhances *S. aureus* colonisation, as a reduction in NMF levels due to abnormal filaggrin metabolism facilitates expression of bacterial clumping factor B, enabling enhanced adhesion to damaged corneocytes [78, 79].

Individual-level interventions may therefore be targeted at restoring microbial diversity on the skin surface. Bacterial diversity may be restored by conventional AD treatments such as emollient and topical corticosteroid use [80, 81]. More novel approaches include cutaneous microbiota transplants and promotion of increased nature-relatedness, whereby microbial diversity is modulated by frequent close contact with nature on an individual level [82–84]. Dog ownership and direct dog exposure, for instance, have been demonstrated in epidemiological studies to confer a protective effect on AD symptom development, likely in a microbiome-dependent fashion but potentially also via modulation of psychological stress levels and neuroinflammation [85, 86].

**Fig. 2:**
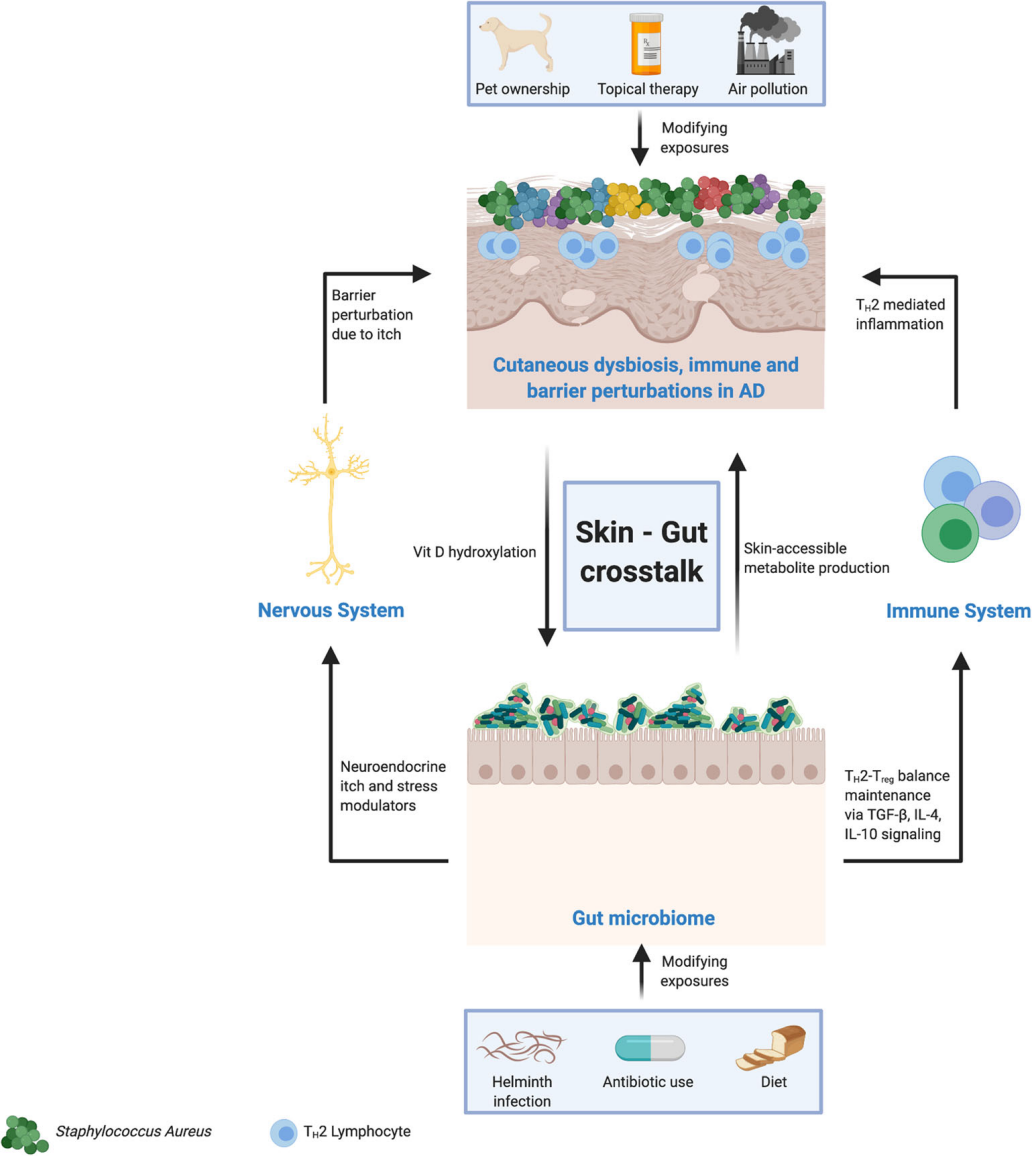
The role of the skin-gut microbiome axis in atopic dermatitis (AD). The skin and gut microbiota are linked via a number of indirect metabolic pathways, with consequent alterations in both microbiome niches. Animal research suggests that certain gut microbial metabolites may be rendered skin-accessible and have an effect on the cutaneous microbiome, whilst the gut microbiome may be modified via a vitamin D hydroxylation in the skin. In addition, gut bacterial dysbiosis has an effect on the skin immune system via a systemic imbalance in the TH2-Treg lymphocyte ratio, further compounding the aberrant type 2 immune response seen in AD. Furthermore, gut bacteria secrete neuroendocrine itch mediators and a dysbiotic gut perpetuates pruritus, further disrupting the skin barrier and facilitating the overgrowth of Staphylococcus aureus in the skin. Both the cutaneous and gut microbiota are susceptible to modification via external exposures, and the timing and dosage of such exposures are important in maintaining a state of health rather than disease

The gut microbiome represents an alternative niche that may be primed for modulation in AD therapy, with a view to ameliorating disease. We have previously discussed a link between the natural environment and gut dysbiosis in AD via a vitamin D–dependent pathway. Infants with AD are more likely to possess a dysbiotic gut microbiome, with a reduction in short-chain fatty acid (SCFA)-producing species (particularly *Bifidobacterium)* and an disproportionate increase in *Bacteroidetes, Firmicutes* and *Proteobacteria* [87]*.* This risk may be compounded by the delivery method, as infants born via caesarean section have demonstrably reduced bacterial diversity at 3 months of age. A recent paper from the EAT study suggested that infants with an increased abundance of *Clostridium census stricto* species in the stool at 3 months are at higher risk of developing AD [88]. In the above study, bacterial diversity and maturation were increased by early introduction of solid foods into the diet, highlighting a potential strategy for gut microbiome manipulation in at-risk infants. Reduced gut bacterial diversity is hypothesised to contribute to AD pathogenesis via regulation of systemic immunity, the so-called skin-gut axis (Fig. 2—gut-skin microbiota crosstalk**.** Bacterial diversity is crucial for the maturation of T_H_1 and T_reg_ pathways, whilst suppressing the aberrant T_H_2-driven immune response that contributes to AD pathogenesis [89, 90]. Current knowledge of the mechanisms underlying skin-gut crosstalk largely focuses on the T_reg_-T_H_2 balance. Experimental evidence from murine models has demonstrated re-programming of T_reg_ cells into T_H_2 cells via reduced TGF-β signalling as a result of expanded innate lymphoid cell 2 (ILC2) populations and IL-4 signalling in the dysbiotic gut [91]. ILC2 cells in skin have also been shown to be expanded in the AD patient population [92]. Conversely, enhanced TGF-β signalling following oral supplementation with *Lactobacillus* strains (which are reduced in the gut microbiome of patients with AD) has been shown to expand the T_reg_ population, thereby contributing to normal age-related maturation of the adaptive immune system [93•]. An alternative hypothesis postulates that alongside regulating systemic immunity, metabolites from gut microbiota and/or the diet are skin accessible [94]. The evidence in support of this hypothesis largely comes from studies on SCFAs. Experimental evidence has demonstrated inhibition of *S. Aureus* growth by propionic acid produced by *Propionibacterium acnes* and topical application of butyrate (normally produced in the gut by SCFA producing species) expanded the local cutaneous T_reg_ population [95, 96]. Early-life oral antibiotic use (which varies significantly between different countries) is associated with a higher prevalence of AD symptoms, likely through their effect on the gut microbiome and the gut-skin microbiome axis. Maternal antimicrobial use during late pregnancy is also associated with higher AD prevalence in offspring [97, 98].

### Dietary factors

The gut microbiome and consequently gut-skin crosstalk and immunity may be amenable to modulation via dietary modification. A Western diet high in trans-fatty acids has been associated with increased AD prevalence in ISAAC Phase One, although the precise mechanism contributing to the above observation remains to be elucidated [99]. Certain elements of the rural lifestyle, particularly consumption of unpasteurised milk, have been linked with reduced prevalence of AD symptoms [100]. Indeed, evidence from the genetically homogenous but socioeconomically disparate region of Karelia, which spans the Finnish-Russian border, demonstrated lower prevalence of allergic disorders in the non-Westernised Russian region, which correlated closely with increased *Acinetobacter* diversity and abundance in populations living in the region [101]. Data from the Promotion of Breastfeeding Intervention Trial (PROBIT) and ISAAC Phase 2 highlighted that breastfeeding (but not prolonged or exclusive breastfeeding) confers a protective effect against AD in early-life [102, 103••, 104]. The mechanisms underpinning the above epidemiological associations may be associated with transfer of microbiota from either unpasteurised milk or breastmilk to the infant, or, alternatively the transfer of soluble immunoactive mediators such as TGF-β and IgA [105–107]. Alongside the type of exposure, timing of exposure is likely to be crucial. Antenatal exposure to farm animals and a maternal diet rich in n-3 poly-unsaturated fatty acids have both been demonstrated to reduce the incidence of AD in the offspring via regulation of foetal immunity and potential microbiotal alterations (e.g. via transplacental passage of microbial metabolites), whilst a diet high in n-6 poly-unsaturated fatty acids had the inverse effect [106, 108, 109]. There is no evidence for dietary probiotic supplementation with *Lactobacillus* and *Bifidobacteria* species in treating established AD. However, their use does appear to reduce the relative risk of AD in offspring when used by women in the last trimester of pregnancy and the infant prior to the development of AD [110, 111••, 112]. The evidence for pre-biotics is currently sparse, with improvements in AD severity demonstrated following dietary supplementation with ketose in one trial of 29 participants, whilst a separate trial of mixed galacto-oligosaccharides found no evidence of symptomatic improvement [113, 114]. A systematic review of pre-biotics for the prevention of AD in infants did not find their effect to be significant for risk reduction [115].

### Psychological and neuromodulatory influences

**Fig. 3:**
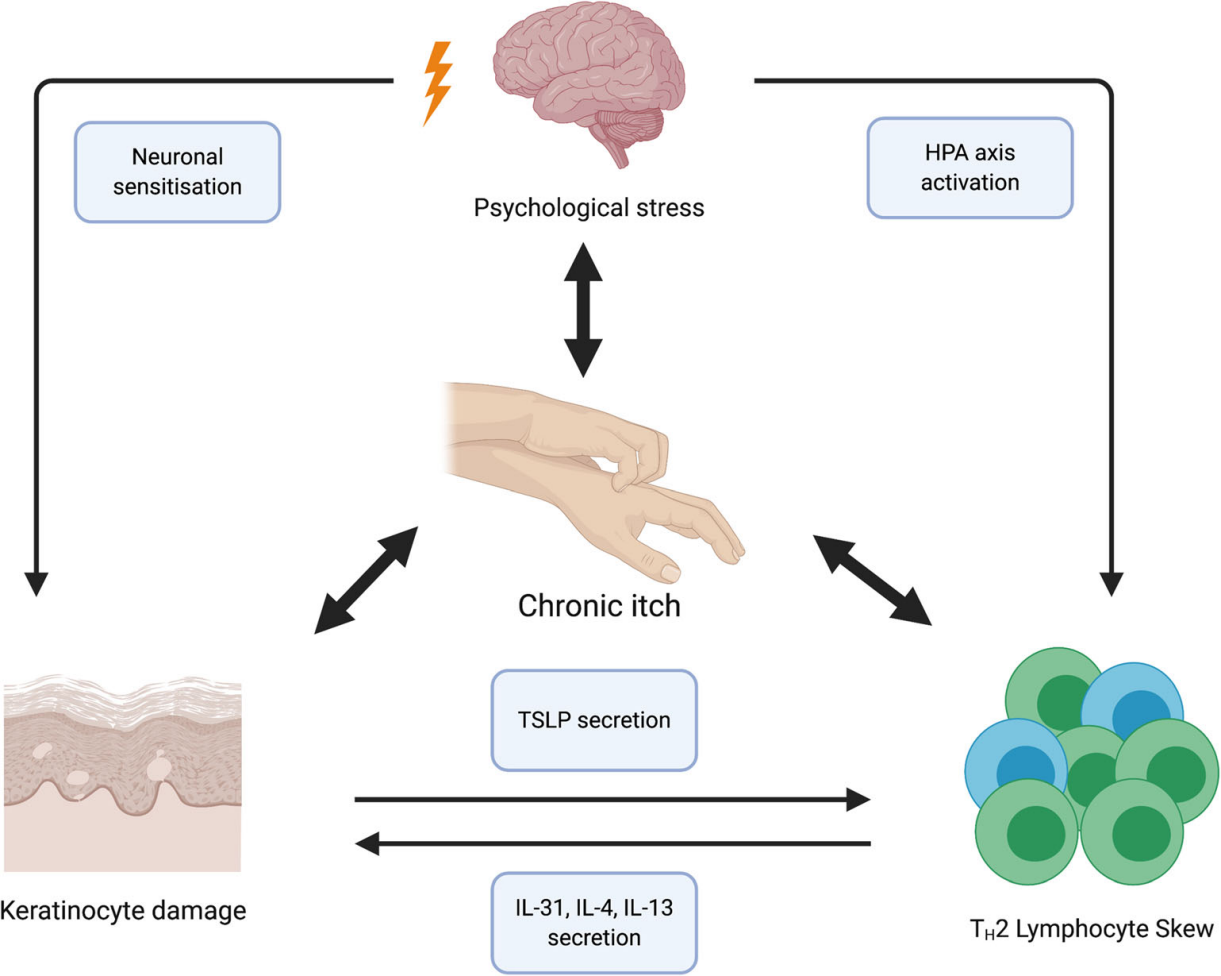
The interplay of the central nervous, immune and cutaneous systems in atopic dermatitis (AD). The multi-directional interactions of the central nervous (CNS), systemic immune systems and the AD lesional skin are complex. Psychological stress may lead to a TH2-dominant peripheral lymphocyte phenotype via activation of the hypothalamic-pituitary-adrenal (HPA) axis. Consequently, the secretion of TH2 cytokines, in particular IL-31, IL-4 and IL-13, damages cutaneous keratinocytes through persistent inflammation, leading to the secretion of thymic stromal lymphopoietin (TSLP) by keratinocytes and further expanding the TH2 population, thereby creating a feed-forward loop. The above pathways are further compounded by the key AD symptom of chronic itch. Chronic itch not only perpetuates psychological distress, often leading to its chronicity and an impact on lifestyle, but ultimately damages the keratinocytes and leads to cutaneous nervous sensitisation, whereby a minimal pruritogenic stimulus produces an excessive pruritic response

There is great inter-individual variability in the neurally mediated responses to psychological stress and subsequent AD flares [116]. The converse also holds true, with recent evidence demonstrating a higher incidence of later life behavioural problems in children with AD [117]. The latter may be explained in part by an immuno-psychiatric hypothesis, stemming from an enhanced understanding of immune processes in regulating CNS homeostasis and individual resilience [118] (Fig. 3—neuroimmune interactions in AD)**.** An overactive immune system is thought to increase brain vulnerability, which in turn, coupled with a “second hit” in later life, manifests as neuropsychiatric disorders. Experimental evidence has linked peripheral T_H_1/T_H_17 lymphocyte subset expansion to the development of psychosis and bipolar affective disorder in later life; however, evidence regarding the neuropsychiatric sequelae of T_H_2-skewed immunity is currently sparse [119, 120]. The biological mechanisms underpinning the response to psychological stress in AD involve a secretion of neuroendocrine modulators and cutaneous pruritoceptor sensitisation leading to a chronic itch-scratch cycle and thus barrier disruption. Psychological stress induces a central stress response via the hypothalamic-pituitary-adrenal (HPA) axis, leading to glucocorticoid and substance P secretion. This consequently translates into a systemic neuro-immune response with resulting T_H_2 skew and mast cell priming [121]. In the epidermis, the T_H_2 skewed immune system acts in concert with keratinocytes to create a feed-forward loop of pruritus, mediated primarily by non-histaminergic pruritoceptive C fibres. Cytokines secreted by T_H_2 cells bind to their respective pruritoceptors on said fibres, as well as exerting the direct stimulating effect of IL-4 and IL-13 on keratinocytes to produce thymic stomal lymphopoietin (TSLP). TSLP consequently feeds-forward, promoting ongoing cytokine secretion by T_H_2 lymphocytes, as well as directly binding to its own pruritoceptor [122].One of the critical T_H_2-secreted itch mediators is IL-31. Its secretion has been demonstrated to induce scratching behaviours in multiple animal models, while IL-31 receptor blockage via monoclonal antibody has been deemed efficacious in ameliorating pruritus in clinical AD trials [123, 124]. A recent mouse model study of AD has also highlighted that photoablation of IL-31 receptors on pruritogenic neurons following selective targeting with a ligand resulted in long term reduction in pruritus and selective retraction of pruritogenic neurons from the skin [125]. The keratinocyte injury as a result of mechanical trauma from scratching induces further inflammatory signalling and thereby amplifies the cycle, often until a state of neuronal sensitisation is attained and even a minimal pruritogenic stimulus results in hyper-acute perception of itch [126•]. The chronic itch-scratch cycle contributes to anxiety, perpetuating psychological distress and resulting in the aforementioned neuro-immune sequelae, alongside further disruption of the skin barrier [116, 127]. The most promising novel therapies aimed at breaking the itch-scratch cycle in AD target primarily the type 2 cytokine and IL-31 signalling pathways and have been reviewed in detail elsewhere [126•]. Emerging evidence also suggests that the gut microbiome may play a role in indirectly modulating the itch-scratch cycle. *Lactobacilli* and *Bifidobacteria* produce γ-aminobutyric acid (GABA)—a central nervous system inhibitory neurotransmitter acting downstream of the pruritoceptive C-fibre pathway, thereby ameliorating skin itch [128]. The gut microbiome is susceptible to alterations in systemic glucocorticoid levels, highlighting the intimate link between the immunobiome, microbiome and psychological stress as a unique individual-specific “internal” exposome domain that may be primed for therapeutic targeting [93•].

The above mechanisms may contribute to a lasting systemic effect, encompassing immune dysregulation, neuropsychiatric comorbidity and AD chronicity. For instance, it is well established that sleep disturbance is prevalent in the AD patient population, affecting up to 60% of individuals [129, 130]. Sleep disturbance may be defined by an imbalance in both sleep continuity (total sleep time, sleep fragmentation) and sleep architecture (the ratio of slow wave sleep (SWS) to rapid eye movement (REM) sleep). A recent review has proposed that sleep disturbance is an immunoregulatory response to stress due to environmental threat, the nature of such disturbance being dependent on the acuity or chronicity of said threat [131]. In the modern environment, the threats encountered by individuals are by and large chronic, psychosocial ones as opposed to more immediately threatening adversities such as predators and/or infectious disease. Patients with AD may have a heightened perception of chronic psychosocial adversity due to their condition, resulting in a limitation of lifestyle and social avoidance as a mechanism for threat evasion [132••]. Sleep disturbance in the chronic stress setting (impaired continuity, decrease in the SWS:REM ratio) has been demonstrated to skew the peripheral immune profile towards a T_H_2 phenotype, further perpetuating the immune dysregulation seen in AD [131]. Managing the psychological component of AD can therefore be viewed as an integral part of the holistic approach to care in an effort to address the perturbed physiological pathways that underpin AD pathogenesis [133].

## Conclusion and future perspectives

Current evidence in AD points towards a sophisticated biological response to environmental exposures at the genetic, immune, microbiome and skin barrier levels. The timing and dosage of exposures is critical when considering modification of environmental factors in primary prevention and therapeutics in AD. Taken together, a holistic exposomal approach requires intervention at the global, community and individual level to ameliorate and potentially prevent disease. While many scientific advances have been made with regard to the nature of the precise biological response to specific environmental triggers, there remain many unknowns and challenges. Modern technology, particularly the use of longitudinal personal monitoring and artificial intelligence, may provide additional insight into the key questions of the “dose-timing response” paradigm [134••, 135]. Identifying the key -omics perturbations of a particular condition and the biomarkers associated with such perturbations may enable clinicians to pinpoint distinct phenotypes within a particular condition, subsequently targeting appropriate prevention and therapeutic pathways and move further into the realm of personalised medicine.

### Availability of data and material

Not applicable.

### Code availability

Not applicable.
